# Tunneling Times in an Asymmetric Harmonic Double-Well with Application to Electron Transfers in Biological Macromolecules

**DOI:** 10.1021/acsomega.4c08622

**Published:** 2024-12-09

**Authors:** João Marcos Costa Monteiro, Elso Drigo Filho

**Affiliations:** Department of Physics, Institute of Biosciences, Humanities and Exact Sciences, São Paulo State University (UNESP), São José do Rio Preto, 15054-000 São Paulo, Brazil

## Abstract

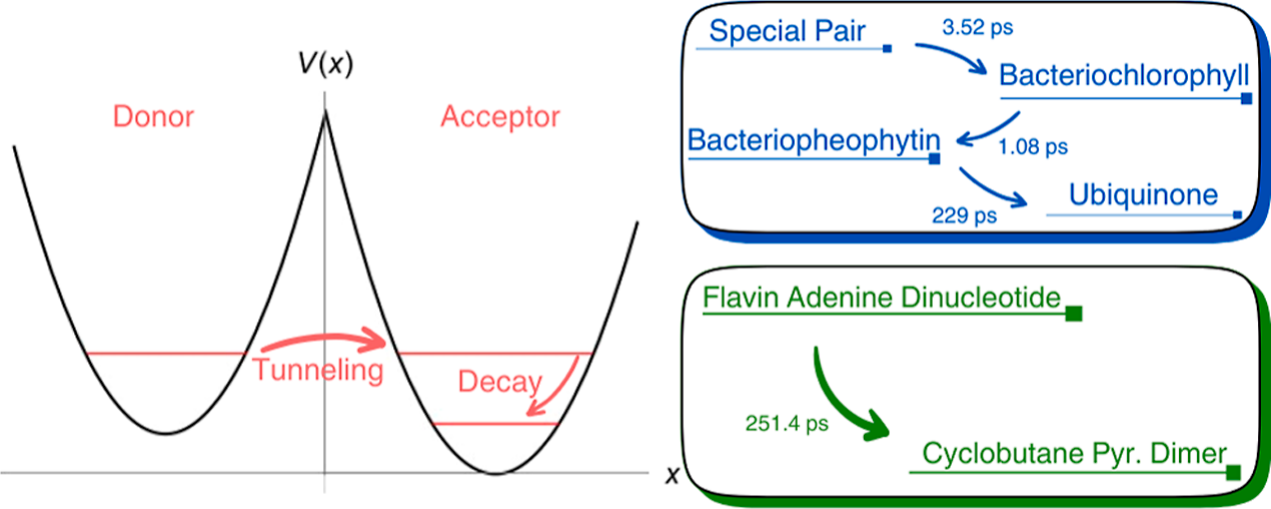

Tunneling times were calculated in electron transfer processes using an asymmetric harmonic double-well model. The simplicity of a direct variational calculation in the approximate solution of the Schrödinger equation, along with the interpretation of tunneling times within the probabilistic framework of a two-level system, allows for the efficient and accurate determination of tunneling times with minimal computational cost. These calculations were applied to electron transfer processes in the study of the photosynthetic reaction center and in the context of catalysis in UV-induced DNA lesion repair and are in agreement with the experimental, computational, and theoretical results with which they were compared. It was seen that the donor–acceptor distance needed to be adjusted for closer agreement between the calculated and experimentally observed times. However, the adjusted values for this distance remain close to those reported in the literature.

## 1 Introduction

Tunneling through a potential barrier is one of the most intriguing and well-explored problems in quantum mechanics.^1^ Since its introduction in alpha decay studies by Gamow,^2^ many aspects of tunneling theory have been developed,^3^ driven by its relevance in the development of several modern technologies such as scanning tunneling microscopy,^4,5^ flash memory,^6^ and quantum computing.^7^

Historically, significant attention from biomolecular researchers has been directed toward electron transfer in cytochrome c,^8−12^ notably highlighted by the work of Chance and DeVault,^13,14^ who postulated that the description of electron transfer in this molecule must be quantum mechanical. Subsequently, experimental studies indicated that the transfer depends on temperature up to a critical point. At lower temperatures, the reaction rate remains constant, leading to the conclusion that under these conditions, electron transfer occurs via quantum tunneling.^14,15^ In addition to its applications in physics, tunneling has been explored in various biomolecular processes, including enzyme kinetics,^16^ photosynthesis,^17^ cellular respiration^18^ and olfaction.^19^

Tunneling often affects reaction rates by enabling electrons or protons to bypass conventional energy barriers during enzymatic catalysis.^20^ In photosynthesis, electron tunneling further facilitates efficient energy transfer within molecular complexes.^21^ See refs (22–24) for recent reviews articles on quantum biology.

One of the oldest challenges in developing theories about quantum tunneling is the determination and interpretation of tunneling times. See the work of Hauge and Støvneng^25^ for a brief review of the various interpretations of tunneling times. Recently, the authors of the current study suggested a method for calculating tunneling times in a bistable harmonic well model,^26^ exploring both symmetric and asymmetric configurations. Here, the model is applied to address biological electron transfers in the two contexts: photosynthetic reaction centers in purple bacteria and the catalysis of UV-induced DNA lesion repair. The central concept of the model is to set the energy levels of the acceptor sites, which values are well-known in the literature,^27−29^ within a double harmonic well framework. Electron transfers in proteins typically have a defined directionality, as they often involve electron hopping between multiple redox centers. Therefore, an asymmetric double-well configuration is more suitable for representing these transfers, as it allows for directional decay after tunneling across the potential barrier. This decay is usually several orders of magnitude faster than tunneling back to the donor site.^26^

To investigate the biological applicability of the model, we first examine electron transfer processes within the photosynthetic reaction centers of purple bacteria, which are responsible for absorbing light to initiate the early stages of photosynthesis. These reaction centers are protein complexes consisting of two subunits, L and M, which have relative 2-fold symmetry between them, where photoactivation occurs, followed by a cascade of electron transfers.^30^ Due to a more favorable arrangement and an asymmetry in the amino acids surrounding the pigments, only the L branch participates in the photosynthetic process.^31,32^ Thus, in this study, we focus only on the tunneling events that occur in this branch. We chose to analyze the applicability of the proposed model by studying the reaction center of the purple bacterium *Rhodobacter sphaeroides* due to the extensive information available in the literature. This aims to better understand the fundamentals of electron transfers in photosynthetic reaction centers. Although there are usually subtle differences in kinetics, distances, and energy levels when studying different bacteria, we believe that the model and the results have general applicability for treating any bacterial reaction center.

After the photoactivation of the special pair (P) of chlorophylls, a sequence of tunneling events occurs between the cofactor arrangement in the L-branch of the *R. sphaeroides* reaction center, initially passing through the bacteriochlorophyll (*B*_L_) and bacteriopheophytin (*H*_L_) within a few picoseconds. Subsequently, the electron is transferred to the quinone (*Q*_L_) in a more prolonged process.^27,33^

This study also explores the biological applicability of the model in DNA photolyases: enzymes responsible for repairing DNA lesions induced by exposure to ultraviolet radiation. One of these lesions consists of the formation of cyclobutane pyrimidine dimers (CPDs) through the cycloaddition of two adjacent thymine bases,^34,35^ contributing to the formation of cancer in humans.^36^ Our focus is on *Anacystis nidulans* DNA photolyase, disregarding the subtle differences in kinetics and energy levels that may arise in the comparison between photolyases obtained from other sources or conditions. The catalysis of the cyclobutane ring cleavage process occurs after photoactivation (absorption usually occurs in the range between 350 and 500 nm) through long-range electron transfer.^37^ Recent studies^37−40^ discuss the mechanism of electron transfer for this system, highlighting models that suggest the influence of the adenine moiety in a first electron transfer via a hopping mechanism, in contrast to other studies^41,42^ that suggest a direct transfer mechanism to the CPD.

Another recent discussion on photolyases concerns the determination of the acceptor site of the first electron transfer. In the electron hopping study,^43^ the interpretation is that the electron is transferred from the fully reduced flavin adenine dinucleotide (*FADH^–^) excited state to the adenine moiety, and then to the distal side (5′) of the T–T dimer. In contrast, density functional theory and ab initio calculations^41^ point to the transfer occurring to the 3′ side of the dimer, as we model here. However, due to the small difference in distances (i.e., the distance between the donor and acceptor sites is practically the same for both 3′ and 5′ CPD), we expect our model to be suitable for either of these transfer pathways.

The rest of the article is organized as follows: Section 2 introduces the potential well model alongside a detailed explanation of the tunneling and decay mechanisms applied to electron transfer processes. Section 3 summarizes the methodology, highlighting the linear combination of the trial wave function for use in the variational principle and the Rabi formula, which describes the oscillation between energy levels in a two-level system. Section 4 presents the results along with discussions on the validation of the model in studying electron transfers in biological macromolecules. Section 4.1 presents more specific results on tunneling times in the reaction center during the initial stages of the photosynthesis process in a purple bacterium, while Section 4.2 investigates the first electron transfer in a photolyase during the repair of ultraviolet-induced lesions in a DNA molecule. Section 5 presents the conclusions.

## 2 Asymmetric Harmonic Double-Well

In the model presented here, each electron transfer is conceptualized as a tunneling process followed by decay within an asymmetric double harmonic well. We fix the energy levels of the acceptor sites, which are well-known in the literature, within an asymmetric harmonic double-well potential and use then in the Schrödinger equation to study the electron transfer processes. This potential is described by eq 1
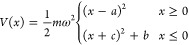
1where *a* and *c* are the equilibrium points of the harmonic wells on the right and left, respectively, and *b* = ℏω is a constant added to the left well to make its ground state resonate with the first excited state of the right well. The equilibrium points *a* and *c* are used as references to determine the donor–acceptor distance, *r*_DA_ = *a* + *c*.

**Figure 1:**
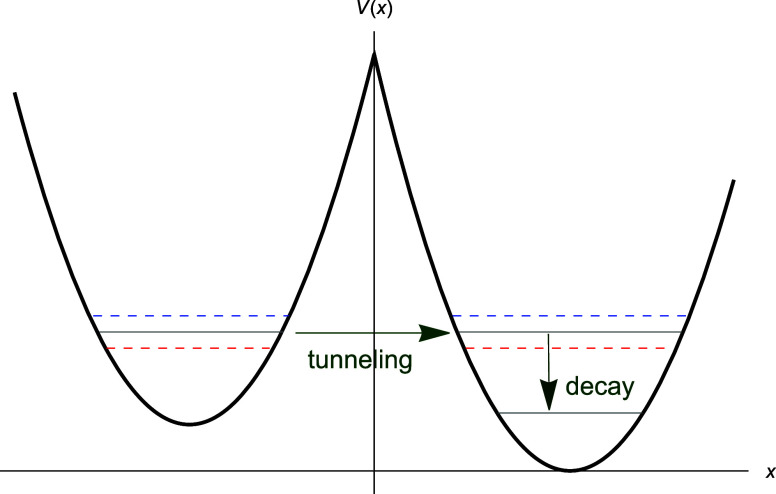
Potential well—eq 1—in atomic units, represented by the solid black line. Parameters are angular frequency ω = 2 and equilibrium positions *a* = 3 and . The horizontal lines illustrate the energy levels as follows: solid gray lines indicate the ground state of the left well ψ_0_^L^ and the first excited state ψ_1_^R^ (upper gray line) and ground state ψ_0_^R^ (bottom gray line) of the right well. The dashed lines represent the energy splitting due to electronic coupling: χ_–_ corresponds to the new ground state (red), and χ_+_ represents the new first excited states (blue).

The eigenvalue of the ground state energy level of the right well is fixed with the values found in the literature and is then used to calculate the angular frequency ω. Since the barrier between the wells is finite, the nearby energy levels interact according to the theory of the two-level system.^44^ Thus, for the proposed model, the interaction between the ground state of the left well (ψ_0_^L^) and the first excited state of the right well (ψ_1_^R^) gives rise to two new energy levels (χ_∓_ with energies *E*_∓_), corresponding to the superposition of the two initial states. Usually, the eigenvalues *E*_∓_ are determined based on a given value of electronic coupling *W*_LR_; however, in this study, *E*_∓_ are estimated variationally, as we do not assume a given value of electronic coupling. In Figure 1, the asymmetric double harmonic well, eq 1, is represented by the solid black line. The energy levels *E*_0_^L^ and *E*_1_^R^ are depicted by solid gray lines, along with the ground state energy level of the right well *E*_0_^R^, which does not interact with the other energy levels. The dashed red and blue lines in Figure 1 correspond to the new energy levels *E*_∓_, respectively.

We assume that the electron initially occupies the ground state of the left well ψ_0_^L^. Due to the interaction with the first excited state of the right well ψ_1_^R^, the electron then occupies the new lower energy state χ_–_. We then impose the maximum transition probability, implying that the electron tunnels through the potential barrier to the right well (horizontal green arrow in Figure 1); the first instance of time at which the probability is 100% is taken as the tunneling time *t*_tun_. Once occupying an orbital in the right well, the electron almost immediately decays to the ground state ψ_0_^R^ (vertical green arrow in Figure 1) and becomes trapped there. This procedure is repeated for each transfer in systems where multiple tunneling events occur (e.g., the photosynthetic system described in Section 4.1).

## 3 Methods

The process to study the asymmetric double oscillator begins with the construction of the trial eigenfunction for the variational principle, written in terms of the eigenfunctions of two isolated oscillators with their respective displaced minima, in the form
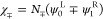
2ψ_0_^L^ corresponds to the ground state of the left well, while ψ_1_^R^ corresponds to the first excited state of the right well and
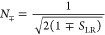
3where *S*_LR_ is the overlap integral of the states ψ_0_^L^ and ψ_1_^R^.

Notably, ψ_0_^L^ and ψ_1_^R^ can be obtained by solving the Schrödinger equation for each well independently, using various solution methods. In particular, we employ the algebraic formalism of supersymmetric quantum mechanics.^26,45^ These eigenfunctions can be written as
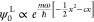
4and
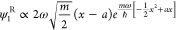
5

**Figure 2:**
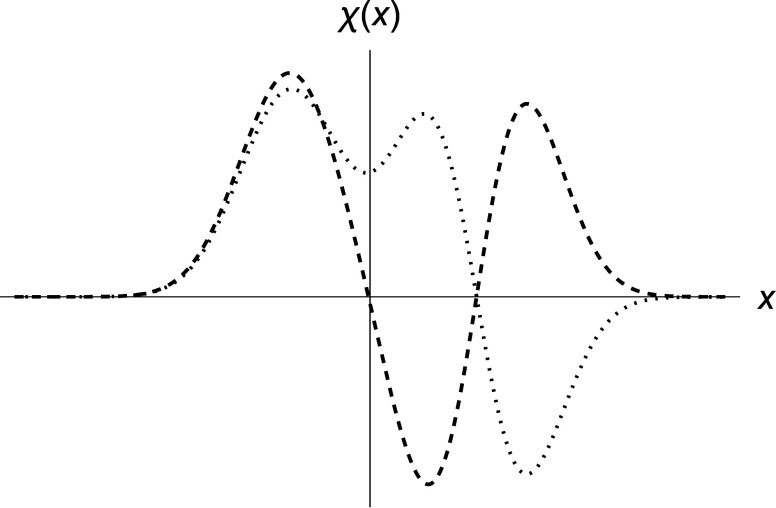
Normalized eigenfunctions χ_–_ (dotted) and χ_+_ (dashed)—eq 2–in atomic units. Parameters are ω = 0.5 and *a* = 3.

The linear combination shown in eq 2 with the eigenfunctions in eqs 4 and 5 consists of a first excited state χ_–_ and a second excited state χ_+_, as mentioned in Section 1, by their respective number of nodes,^46^ as shown in Figure 2.

The eigenfunctions χ_∓_ are then used in the variational principle to determine the eigenvalues *E*_∓_
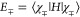
6Equation 6 can be used to calculate the value of the electronic coupling *W*_LR_ through the two-level system theory. Since the states represented by the eigenfunctions ψ_0_^L^ and ψ_1_^R^ are resonant, it follows that *E*_∓_ = *E*_0_^L^ ∓ *W*_LR_.

Since the Hamiltonian *H* does not explicitly depend on time, we can obtain the temporal evolution vector of the system (for a given initial condition) in terms of ψ_0_^L^ and ψ_1_^R^. The electron transition probability between the wells can be described by the Rabi formula^44^
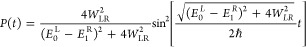
7

By considering that the energy levels are resonant and imposing the maximum probability *P* = 1, which is achieved when the sine function reaches its maximum, the tunneling times *t*_tun_ can be calculated as
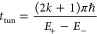
8where *k* is an integer. Imposing the maximum transition probability ensures that the electron is transferred between the wells, having already been used for the calculation of tunneling times in square wells for the study of photosynthetic systems.^47^

## 4 Results and Discussion

### 4.1 Photosynthetic Reaction Centers of *R. sphaeroides*

As described in Section 2, at each transfer step we set the conditions such that the ground state of the right well (ψ_0_^D^, with eigenvalue *E*_0_^D^) equals the value displayed in the literature,^27,28^ allowing us to estimate a fixed value for ω through the well-known result of the energy eigenvalue of the harmonic oscillator, The values of ω for the steps *P → *B*_L_, *B*_L_ → *H*_L_, and *H*_L_ → *Q*_L_ are shown in Table 1.

**Table 1:** Comparison between the Calculated Tunneling Times *t*_tun_ and Experimental Tunneling Times Reported in the Literature^27,33,48,49^ for *R. Sphaeroides* Reaction Centers

ET step	ω (s^–1^)	*t*_tun_ (ps)	*t*_tun_ (ps)^27^	*t*_tun_ (ps)^33^	*t*_tun_ (ps)^48^	*t*_tun_ (ps)^49^
*P → *B*_L_	3.95 × 10^15^	3.52	≈3.0	3.5 ± 0.4	3.5 ± 0.4	3–4
*B*_L_ → *H*_L_	3.50 × 10^15^	1.08	≈0.9	0.9 ± 0.3	1.2 ± 0.3	<1
*H*_L_ → *Q*_L_	1.97 × 10^15^	229	≈200	220 ± 40	—	200

After determining ω, the potential well can be constructed, and the methodology outlined in Section 3 was applied to determine the tunneling times of each step, as also shown in Table 1.

The results of this study were compared with ultrafast transient absorption spectroscopy results.^33,48^ The comparisons show that the calculated tunneling times values obtained falls within the reported experimental error margins. The analyses are also consistent with more recent studies using different analysis techniques,^27,49^ reinforcing the reliability of our results. Notably, the largest experimental error occurs in the final step, as the electron transfer is directed toward a more flexible region of the reaction center, where dynamic factors exert a greater influence. These factors include molecular motion due to temperature fluctuations and pressure conditions, as well as inhomogeneous solvent relaxation.^50^ These influences, which are not accounted for in the model, are expected to directly affect electronic coupling and significantly impact the reaction rate.^15^ In this context, there is a greater discrepancy between the calculated value of 229 ps and the experimental values shown in Table 1. Furthermore, the quinone (*Q*_L_) is smaller in size compared to the other cofactors, resulting in fewer unoccupied electronic orbitals and longer tunneling times. Zabelin et al.^51^ analyzed the electron transfer chain by dividing it into two stages: *P → *H*_L_ and *H*_L_ → *Q*_L_. Their study considers reaction centers under different conditions, but our comparison is limited to the results reported for aqueous solution. For this condition, the estimated characteristic electron transition times are 3.6 and 197 ps, respectively. Our calculated times align closely with the first step and remain of the same order of magnitude for the second step. Our results are also in agreement with the time constants obtained through the hopping model (multistep electron tunneling),^52^ particularly as the agreement between our results and their treatment of the first two transfers (*P → *B*_L_ → *H*_L_) as a hopping mechanism and the last electron transfer as single step tunneling.

Due to the adjustment in the equilibrium points of the harmonic wells, which was necessary to obtain accurate results for *t*_tun_, an analysis of the impact of this adjustment on the donor–acceptor distance *r*_DA_ should be conducted. Table 2 shows the distances used in this study and compares them with experimental results obtained through X-ray diffraction^53,54^ and with results estimated from the positions of the center of mass of the cofactors.^55^ Our results fall within the experimental uncertainty^53^ for steps *P → *B*_L_ and *B*_L_ → *H*_L_. However, for the last tunneling, the donor–acceptor distance is outside the experimental margin of error, which we attribute to the thermal dependence of this tunneling step.^50,56^ In general, the distances involved in electron transfer processes in biology vary between the theoretical models, due to electron delocalization in aromatic rings. So, relatively shorter distances can be considered, including the closest distances (edge-to-edge distances) reported by Warren et al.,^43,52^ giving better agreement between calculated time constants and experimental data.

**Table 2:** Donor–Acceptor Distances *r*_DA_ for Each Step of Electron Transfer in *R. sphaeroides* Reaction Centers

ET step	*r*_DA_ (Å)	*r*_DA_ (Å)^53,54^	*r*_DA_ (Å)^55^
*P → *B*_L_	11.2	11.0 ± 0.4	10.6
*B*_L_ → *H*_L_	11.4	10.5 ± 0.4	10.9
*H*_L_ → *Q*_L_	18.8	13.0 ± 0.4	13.8

### 4.2 DNA Photolyase Repair Mechanism

In this study we addressed the electron transfer in DNA photolyase as single step reaction. Our calculations for the tunneling time considering the adenine intermediate (*FADH^–^ → A^–^) are of the order of 10^–8^ s for *r*_DA_ = 11 Å, which is slower in this step than the reality reported in experiments^39^ for the role process. Treating the transfer as a single step (*FADH^–^ →3^′^-T CPD) allows the calculation of *t*_tun_ consistent with experimental observations while maintaining reasonable distances *r*_DA_. The fluorescence spectrum of DNA Photolyase^29^ estimates an energy of 4.165 × 10^–19^ J (2.6 eV) for the excited state *FADH^–^. According to Warren et al.,^43^ the energy difference between * FADH^–^ and the T–T dimer is of the order of 0.5 ± 0.1 eV (8.01 × 10^–20^ J). Thus, we set the ground state of the right well (acceptor) with an energy of 3.365 × 10^–19^ J, allowing us to estimate ω = 6.38 × 10^15^ s^–1^. Constructing the potential with the estimated value of ω and proceeding with the methodology described in Section 3, a value of 251.4 ps is calculated for *t*_tun_, which is displayed in Table 3 and compared with other experimental and theoretical studies.

**Table 3:** Comparison between the Calculated Tunneling Times *t*_tun_ and the Experimental^37,39,40^ and Theoretical^41,43^ Tunneling Times Reported in the Literature for the *A. nidulans* DNA Photolyase Catalysis of CPD Dimers

*t*_tun_ (ps)	*t*_tun_ (ps)^37,39^	*t*_tun_ (ps)^40^	*t*_tun_ (ps)^41^	*t*_tun_ (ps)^43^
251.4	250^a^	209	230^b^	370

a Result for T–T CPD.

b Study of *Escherichia coli* DNA photolyase.

As seen in Table 3, the calculated results for the electron transfer process are consistent with experimental results for the forward electron transfer obtained through femtosecond-resolved transient-absorption spectroscopy.^37,39,40^ In comparing our theoretical results with those in Table 3, we observe significant differences in calculated electron transfer times. Although these calculations remain within the picosecond scale, consistent with experimental observations, the discrepancies arise from the unique assumptions and methodologies considered by each model. Our study focuses on fundamental aspects of quantum mechanics and calculates tunneling times that closely match experimental values by assuming a single-step electron transfer. By contrast, Warren et al. construct hopping maps^43^ based on differential equations for reaction rates, with an assumed intermediate state in the adenine region of FADH. Their approach includes solvent relaxation dynamics and calculates electronic coupling via the packing density method. On the other hand, Prytkova et al. employ detailed computational simulations,^41^ assuming direct transfer to the dimer and calculating electronic coupling by analyzing unoccupied molecular orbitals. This approach captures an atomistic level description of the protein structure but requires substantial computational power. These approaches exemplify the diversity of models and the need for model-specific adjustments to better align with experimental data. Each method requires careful selection of the most relevant dynamic factors to accurately reflect experimental observations, underscoring the complexity of modeling electron transfer times in biological systems, where a range of influential factors must be considered.

Our results, in particular, demonstrate that describing electron transfers through the mechanism of tunneling and decay within an asymmetric harmonic double-well, though simplified, yields tunneling times that closely approximate experimental values when combined with a careful selection of *r*_DA_ distances. Regarding these distances, we make the same comparison as in the previous section, comparing the donor–acceptor distance *r*_DA_ with values from crystallized structures and values used in other approaches. In this study, the donor–acceptor distance is 10.9 Å, showing a minor discrepancy of 0.8 Å compared to the 11.7 Å distance considered by Warren et al.^43^ Our result also aligns closely with the 11 Å distance obtained by Weber et al. using Density Functional Theory simulations^38^ for *E. coli* photolyase, differing only by 0.1 Å.

Compared with experimental studies summarized in Table 3, Liu et al.^37^ report an average distance for forward electron transfer ranging from 8.0 to 8.2 Å, resulting in a difference of 3.9–3.7 Å, respectively. Furthermore, in the crystal structure of photolyase bound to a CPD-like DNA lesion reported by Mees et al.,^30^ the total transfer pathway distance toward the 3′ CPD is 8.3 Å (8.2 Å toward the 5′ CPD), yielding a discrepancy of 2.6 Å (2.7 Å) relative to our calculated distances.

Among our results, a noticeable contrast emerges in the alignment of the calculated tunneling times and *r*_DA_ distances for the longer charge transfer processes. In Section 4.1, we observed a tunneling time that deviates by 9–29 ps from experimental findings, accompanied by a donor–acceptor distance that differs by more than 5 Å. In contrast, the electron transfer discussed in Section 4.2, while also involving tunneling on the order of 200 ps, exhibits significantly better agreement with experimental data, both in terms of tunneling time and *r*_DA_ distance reported in the literature.

As mentioned earlier, the *H*_L_ → *Q*_L_ transfer in bacterial reaction centers is highly thermally dependent,^50,56^ whereas the molecular arrangement during CPD repair is characterized by a notable rigidity^35^ that likely minimizes fluctuations that could otherwise influence the tunneling mechanism. This observation reinforces the suitability of the proposed model for calculating tunneling times even in long-time electron transfer processes. However, as a limitation of its current formulation, the model achieves its highest numerical accuracy in rigid protein regions, where structural stability enhances the predictive power of the tunneling and decay framework.

## 5 Conclusions

Overall, this study examined the suitability of an asymmetric bistable harmonic potential model to study electron transport in dynamic processes within biological systems. Specifically, characteristic tunneling times for the early stages of photosynthesis and forward electron transfer in DNA photolyase catalysis were determined.

The results were obtained through the study of the Schrödinger equation and the well-established two-level system theory, without the need for more complex methodologies. The only parameter adjusted in our study was the distance between the equilibrium points of the donor and acceptor wells, allowing for tunneling time results that are very close to those observed experimentally while remaining compatible with the distances estimated in experimental and computational studies.

In conclusion, the accuracy of the results and the simplicity of both the model and the calculations suggests that further refinements and extensions of the model for studying other electron transfer systems in biomolecules are worthy of further investigation.

## Supporting Information Available

Computational protocol detailing calculations for each electron transfer; containing the implemented calculations (ZIP)

## References

[ref1] Merzbacher E. The Early History of Quantum Tunneling. Phys. Today 2002, 55, 44–49. 10.1063/1.1510281.

[ref2] Gamow G. The Quantum Theory of Nuclear Disintegration. Nature 1928, 122, 805–806. 10.1038/122805b0.

[ref3] Razavy M. Quantum Theory Of Tunneling, 2nd ed.; World Scientific Publishing Company, 2013.

[ref4] Salmeron M.; Eren B. High-Pressure Scanning Tunneling Microscopy. Chem. Rev. 2021, 121, 962–1006. 10.1021/acs.chemrev.0c00429. 33290057

[ref5] Yang Z.; Freund H.-J. High-speed scanning tunneling microscope technique and its application in studying structural dynamics on surfaces. Prog. Surf. Sci. 2024, 99, 100744 10.1016/j.progsurf.2024.100744.

[ref6] Vavrenyuk A. B.; Makarov V. V.; Shurygin V. A. Flash Memory—Formation, Development and Prospects. In Advanced Technologies in Robotics and Intelligent Systems; Misyurin S., Arakelian V., Avetisyan A., Eds.; Mechanisms and Machine Science; Springer: Cham, 2020; Vol. 80, pp 45–53. 10.1007/978-3-030-33491-8_5.

[ref7] Cai R.; Žutić I.; Han W. Superconductor/Ferromagnet Heterostructures: A Platform for Superconducting Spintronics and Quantum Computation. Adv. Quantum Technol. 2023, 6, 2200080 10.1002/qute.202200080.

[ref8] Hodges H. L.; Holwerda R. A.; Gray H. B. Kinetic studies of the reduction of ferricytochrome c by ethylenediaminetetraacetatoiron(II). J. Am. Chem. Soc. 1974, 96, 3132–3137. 10.1021/ja00817a019. 4364803

[ref9] Gray H. B.; Winkler J. R. Electron Transfer in Proteins. Annu. Rev. Biochem. 1996, 65, 537–561. 10.1146/annurev.bi.65.070196.002541. 8811189

[ref10] Marcus R. A. Electron transfer reactions in chemistry theory and experiment. J. Electroanal. Chem. 1997, 438, 251–259. 10.1016/S0022-0728(97)00091-0.

[ref11] Stock D.; Leslie A. G. W.; Walker J. E. Molecular Architecture of the Rotary Motor in ATP Synthase. Science 1999, 286, 1700–1705. 10.1126/science.286.5445.1700. 10576729

[ref12] Xin H.; Sim W. J.; Namgung B.; Choi Y.; Li B.; Lee L. P. Quantum biological tunnel junction for electron transfer imaging in live cells. Nat. Commun. 2019, 10, 3245 10.1038/s41467-019-11212-x. 31324797 PMC6642182

[ref13] Chance B.; Nishimura M. On the mechanism of chlorophyll-cytochrome interaction: the temperature insensitivity of light-induced cytochrome oxidation in chromatium. Proc. Natl. Acad. Sci. U.S.A. 1960, 46, 19–24. 10.1073/pnas.46.1.19. 16590592 PMC285002

[ref14] DeVault D.; Chance B. Studies of Photosynthesis Using a Pulsed Laser: I. Temperature Dependence of Cytochrome Oxidation Rate in Chromatium. Evidence for Tunneling. Biophys. J. 1966, 6, 825–847. 10.1016/S0006-3495(66)86698-5. 5972381 PMC1368046

[ref15] Devault D. Quantum mechanical tunnelling in biological systems. Q. Rev. Biophys. 1980, 13, 387–564. 10.1017/S003358350000175X. 7015406

[ref16] Schwartz S. D. Protein Dynamics and Enzymatic Catalysis. J. Phys. Chem. B 2023, 127, 2649–2660. 10.1021/acs.jpcb.3c00477. 36944023 PMC10072970

[ref17] Gerodias K. M.; Bernido M. V. C.; Bernido C. C. Resonant tunneling in natural photosynthetic systems. Phys. Scr. 2021, 96, 125038 10.1088/1402-4896/ac3c58.

[ref18] Hayashi T.; Stuchebrukhov A. A. Electron tunneling in respiratory complex I. Proc. Natl. Acad. Sci. U.S.A. 2010, 107, 19157–19162. 10.1073/pnas.1009181107. 20974925 PMC2984193

[ref19] Solov’yov I. A.; Chang P.-Y.; Schulten K. Vibrationally assisted electron transfer mechanism of olfaction: myth or reality?. Phys. Chem. Chem. Phys. 2012, 14, 13861–13871. 10.1039/c2cp41436h. 22899100 PMC3478898

[ref20] Mostajabi Sarhangi S.; Matyushov D. V. Electron Tunneling in Biology: When Does it Matter?. ACS Omega 2023, 8, 27355–27365. 10.1021/acsomega.3c02719. 37546584 PMC10399179

[ref21] Chain R. K.; Arnon D. I. Quantum efficiency of photosynthetic energy conversion. Proc. Natl. Acad. Sci. U.S.A. 1977, 74, 3377–3381. 10.1073/pnas.74.8.3377. 20627 PMC431568

[ref22] Lambert N.; Chen Y.-N.; Cheng Y.-C.; Li C.-M.; Chen G.-Y.; Nori F. Quantum biology. Nat. Phys. 2013, 9, 10–18. 10.1038/nphys2474.

[ref23] Brookes J. C. Quantum effects in biology: golden rule in enzymes, olfaction, photosynthesis and magnetodetection. Proc. R. Soc. A 2017, 473, 20160822 10.1098/rspa.2016.0822. 28588400 PMC5454345

[ref24] Cao J.; Cogdell R. J.; Coker D. F.; Duan H. G.; Hauer J.; Kleinekathöfer U.; Jansen T. L. C.; Mančal T.; Miller R. J. D.; Ogilvie J. P.; et al. Quantum biology revisited. Sci. Adv. 2020, 6, eaaz4888 10.1126/sciadv.aaz4888. 32284982 PMC7124948

[ref25] Hauge E. H.; Støvneng J. A. Tunneling times: a critical review. Rev. Mod. Phys. 1989, 61, 917–936. 10.1103/RevModPhys.61.917.

[ref26] Monteiro J. M. C.; Drigo Filho E. Tunneling times of an electron in one-dimensional symmetric and asymmetric harmonic double-well potentials. Eur. Phys. J. Plus 2023, 138, 718 10.1140/epjp/s13360-023-04364-9.

[ref27] Zinth W.; Wachtveitl J. The First Picoseconds in Bacterial Photosynthesis—Ultrafast Electron Transfer for the Efficient Conversion of Light Energy. ChemPhysChem 2005, 6, 871–880. 10.1002/cphc.200400458. 15884069

[ref28] Katilius E.; Lbabendure J.; Lin S.; Woodbury N. Electron Transfer Dynamics in Rhodobacter sphaeroides Reaction Center Mutants with a Modified Ligand for the Monomer Bacteriochlorophyll on the Active Side. Photosynth. Res. 2004, 81, 165–180. 10.1023/b:pres.0000035048.10358.90.

[ref29] Kao Y.-T.; Saxena C.; Wang L.; Sancar A.; Zhong D. Direct observation of thymine dimer repair in DNA by photolyase. Proc. Natl. Acad. Sci. U.S.A. 2005, 102, 16128–16132. 10.1073/pnas.0506586102. 16169906 PMC1283438

[ref30] Mees A.; Klar T.; Gnau P.; Hennecke U.; Eker A. P. M.; Carell T.; Essen L.-O. Crystal Structure of a Photolyase Bound to a CPD-Like DNA Lesion After in Situ Repair. Science 2004, 306, 1789–1793. 10.1126/science.1101598. 15576622

[ref31] Bylina E. J.; Kirmaier C.; McDowell L.; Holten D.; Youvan D. C. Influence of an amino-acid residue on the optical properties and electron transfer dynamics of a photosynthetic reaction centre complex. Nature 1988, 336, 182–184. 10.1038/336182a0.

[ref32] Nagarajan V.; Parson W. W.; Davis D.; Schenck C. C. Kinetics and free energy gaps of electron-transfer reactions in Rhodobacter sphaeroides reaction centers. Biochemistry 1993, 32, 12324–12336. 10.1021/bi00097a008. 8241119

[ref33] Holzapfel W.; Finkele U.; Kaiser W.; Oesterhelt D.; Scheer H.; Stilz H. U.; Zinth W. Initial electron-transfer in the reaction center from Rhodobacter sphaeroides. Proc. Natl. Acad. Sci. U.S.A. 1990, 87, 5168–5172. 10.1073/pnas.87.13.5168. 11607090 PMC54283

[ref34] Carell T.; Burgdorf L. T.; Kundu L. M.; Cichon M. The mechanism of action of DNA photolyases. Curr. Opin. Chem. Biol. 2001, 5, 491–498. 10.1016/S1367-5931(00)00239-8. 11578921

[ref35] Sancar A. Structure and Function of DNA Photolyase and Cryptochrome Blue-Light Photoreceptors. Chem. Rev. 2003, 103, 2203–2238. 10.1021/cr0204348. 12797829

[ref36] Taylor J. S. Unraveling the Molecular Pathway from Sunlight to Skin Cancer. Acc. Chem. Res. 1994, 27, 76–82. 10.1021/ar00039a003.

[ref37] Liu Z.; Tan C.; Guo X.; Kao Y.-T.; Li J.; Wang L.; Sancar A.; Zhong D. Dynamics and mechanism of cyclobutane pyrimidine dimer repair by DNA photolyase. Proc. Natl. Acad. Sci. U.S.A. 2011, 108, 14831–14836. 10.1073/pnas.1110927108. 21804035 PMC3169159

[ref38] Weber S.; Möbius K.; Richter G.; Kay C. W. M. The Electronic Structure of the Flavin Cofactor in DNA Photolyase. J. Am. Chem. Soc. 2001, 123, 3790–3798. 10.1021/ja003426m. 11457111

[ref39] Liu Z.; Guo X.; Tan C.; Li J.; Kao Y.-T.; Wang L.; Sancar A.; Zhong D. Electron Tunneling Pathways and Role of Adenine in Repair of Cyclobutane Pyrimidine Dimer by DNA Photolyase. J. Am. Chem. Soc. 2012, 134, 8104–8114. 10.1021/ja2105009. 22533849 PMC3354007

[ref40] Zhang M.; Wang L.; Zhong D. Photolyase Dynamics and electron-transfer mechanisms of DNA repair. Arch. Biochem. Biophys. 2017, 632, 158–174. 10.1016/j.abb.2017.08.007. 28802828 PMC5650541

[ref41] Prytkova T. R.; Beratan D. N.; Skourtis S. S. Photoselected electron transfer pathways in DNA photolyase. Proc. Natl. Acad. Sci. U.S.A. 2007, 104, 802–807. 10.1073/pnas.0605319104. 17209014 PMC1783394

[ref42] Acocella A.; Jones G. A.; Zerbetto F. What Is Adenine Doing in Photolyase?. J. Phys. Chem. B 2010, 114, 4101–4106. 10.1021/jp101093z. 20184295

[ref43] Warren J. J.; Ener M. E.; Vlček A.; Winkler J. R.; Gray H. B. Electron hopping through proteins. Coord. Chem. Rev. 2012, 256, 2478–2487. 10.1016/j.ccr.2012.03.032. 23420049 PMC3570191

[ref44] Cohen-Tannoudji C.; Diu B.; Laloë F. Quantum Mechanics; Wiley, 1977.

[ref45] Cooper F.; Khare A.; Sukhatme U. Supersymmetry and quantum mechanics. Phys. Rep. 1995, 251, 267–385. 10.1016/0370-1573(94)00080-M.

[ref46] Merzbacher E. Quantum Mechanics; Wiley, 1970.

[ref47] Drigo Filho E.; Jubilato K. H. P.; Ricotta R. M. Photoinduced Quantum Tunneling Model Applied to an Organic Molecule. Braz. J. Phys. 2020, 50, 575–581. 10.1007/s13538-020-00782-7.

[ref48] Lauterwasser C.; Finkele U.; Scheer H.; Zinth W. Temperature dependence of the primary electron transfer in photosynthetic reaction centers from Rhodobacter sphaeroides. Chem. Phys. Lett. 1991, 183, 471–477. 10.1016/0009-2614(91)80161-P.

[ref49] Magdaong N. C. M.; Faries K. M.; Buhrmaster J. C.; Tira G. A.; Wyllie R. M.; Kohout C. E.; Hanson D. K.; Laible P. D.; Holten D.; Kirmaier C. High Yield of B-Side Electron Transfer at 77 K in the Photosynthetic Reaction Center Protein from Rhodobacter sphaeroides. J. Phys. Chem. B 2022, 126, 8940–8956. 10.1021/acs.jpcb.2c05905. 36315401

[ref50] Wang H.; Lin S.; Allen J. P.; Williams J. C.; Blankert S.; Laser C.; Woodbury N. W. Protein Dynamics Control the Kinetics of Initial Electron Transfer in Photosynthesis. Science 2007, 316, 747–750. 10.1126/science.1140030. 17478721

[ref51] Zabelin A. A.; Khristin A. M.; Shkuropatova V. A.; Khatypov R. A.; Shkuropatov A. Y. Primary electron transfer in Rhodobacter sphaeroides R-26 reaction centers under dehydration conditions. Biochim. Biophys. Acta, Bioenerg. 2020, 1861, 148238 10.1016/j.bbabio.2020.148238. 32533935

[ref52] Warren J. J.; Winkler J. R.; Gray H. B. Hopping maps for photosynthetic reaction centers. Coord. Chem. Rev. 2013, 257, 165–170. 10.1016/j.ccr.2012.07.002. 23275678 PMC3530024

[ref53] Allen J. P.; Feher G.; Yeates T. O.; Komiya H.; Rees D. C. Structure of the reaction center from Rhodobacter sphaeroides R-26: the cofactors. Proc. Natl. Acad. Sci. U.S.A. 1987, 84, 5730–5734. 10.1073/pnas.84.16.5730. 3303032 PMC298936

[ref54] Camara-Artigas A.; Brune D.; Allen J. P. Interactions between lipids and bacterial reaction centers determined by protein crystallography. Proc. Natl. Acad. Sci. U.S.A. 2002, 99, 11055–11060. 10.1073/pnas.162368399. 12167672 PMC123209

[ref55] Ermler U.; Fritzsch G.; Buchanan S. K.; Michel H. Structure of the photosynthetic reaction centre from Rhodobacter sphaeroides at 2.65 å resolution: cofactors and protein-cofactor interactions. Structure 1994, 2, 925–936. 10.1016/S0969-2126(94)00094-8. 7866744

[ref56] Guo Z.; Lin S.; Xin Y.; Wang H.; Blankenship R. E.; Woodbury N. W. Comparing the Temperature Dependence of Photosynthetic Electron Transfer in Chloroflexus aurantiacus and Rhodobactor sphaeroides Reaction Centers. J. Phys. Chem. B 2011, 115, 11230–11238. 10.1021/jp204239v. 21827152

