# Supplementary material for: Tunneling Times in an Asymmetric Harmonic Double-Well with Application to Electron Transfers in Biological Macromolecules
Source: ACS Omega. 2024 Dec 9;9(50):49832–8. doi: 10.1021/acsomega.4c08622 (PMC11656229; doi:10.1021/acsomega.4c08622)
Supplement: Supplementary file 1 — ao4c08622_si_001.zip [file ao4c08622_si_001.zip › Supporting Information/Supporting Information.pdf]

Supporting Information

Tunneling Times in an Asymmetric Harmonic Double-Well with Application to Electron Transfers in Biological Macromolecules

João Marcos Costa Monteiro* and Elso Drigo Filho

Department of Physics, São Paulo State University (UNESP), Institute of Biosciences, Humanities and Exact Sciences, São José do Rio Preto, SP, 15054-000, Brazil.

Contents

1. *Rhodobacter sphaeroides* reaction centers

$P^* \rightarrow B_L$ 2

$B_L \rightarrow H_L$ 6

$H_L \rightarrow Q_L$ 10

2. *Anacystis nidulans* DNA photolyase

Single-step $FADH^* \rightarrow 3'CPD$ 14

Bacterial PRC: P to BL

```
(*Defining the relevant physical constants (SI units):*)

In[•]:= ℏ = 1.0545 * 10^(-34)
m = 9.1093 * 10^(-31)
ω = 3.95 * 10^(15)
a = 6 * 10^(-10)
b = ℏ * ω
c = Sqrt[a^2 - (2 * ℏ / (m * ω))]
```

Out[•]= 1.0545×10^{-34}

Out[•]= 9.1093×10^{-31}

Out[•]= 3.95×10^{15}

Out[•]= $\frac{3}{5\,000\,000\,000}$

Out[•]= 4.16528×10^{-19}

Out[•]= 5.48987×10^{-10}

```
(*Defining the bistable potential:*)

In[•]:= V[x_] := Piecewise[{{(0.5) * (m * ω^2) * (x + c)^2 + b, x ≤ 0}, {((0.5) * m * ω^2) * (x - a)^2, x ≥ 0}}]

(*Graphically checking the form of the double well:*)

In[•]:= Plot[V[x], {x, -12 * 10^(-10), 12 * 10^(-10)}]
```

Out[•]=

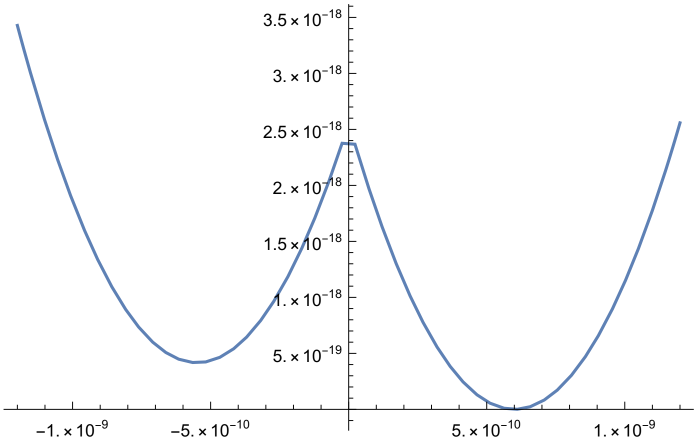


```
(*Comparing the energy values of simple
 oscillators with the height of the potential barrier:*)
```

In[ ]:= NumberForm[E0R = ħ * ω / 2, 9]   (*right well*)
NumberForm[E1R = 3 * ħ * ω / 2 , 9]   (*right well*)
NumberForm[E0L = ħ * ω / 2 + ħ * ω, 9] (*left well*)

Out[ ]//NumberForm=
$2.0826375 \times 10^{-19}$

Out[ ]//NumberForm=
$6.2479125 \times 10^{-19}$

Out[ ]//NumberForm=
$6.2479125 \times 10^{-19}$

In[ ]:= V[0] (*potential barrier*)

Out[ ]= 2.5583×10^{-18}

(*Defining the eigenfunctions of simple harmonic oscillators:*)

In[ ]:= ψ0R[x_] :=

$$\left(1 \Big/ \text{Sqrt}\left[\frac{e^{\frac{m*\omega*a^2}{\hbar}} * \sqrt{\pi}}{\sqrt{\frac{m*\omega}{\hbar}}}\right]\right) * \text{Exp}[(m*\omega/\hbar)*(-x^2/2 + a*x)]$$ (*ground-state - right well*)

In[ ]:= Abs[Integrate[ψ0R[x] * ψ0R[x], {x, -Infinity, Infinity}]]
(*Checking the eigenfunction normalization:*)

Out[ ]= 1.

$$\psi 1R[x\_] := \left(1 \Big/ \text{Sqrt}\left[\frac{e^{\frac{m*\omega*a^2}{\hbar}} * m * \sqrt{\pi} * \omega^2}{\left(\frac{m*\omega}{\hbar}\right)^{3/2}}\right]\right) * 2 * \omega * \text{Sqrt}[m/2] * (x - a) *$$

Exp[(m * ω / ħ) * (-x ^ 2 / 2 + a * x)] (*first excitited state - right well*)

In[ ]:= Abs[Integrate[ψ1R[x] * ψ1R[x], {x, -Infinity, Infinity}]]
(*Checking the eigenfunction normalization:*)

Out[ ]= 1.

In[ ]:= ψ0L[x_] :=

$$\left(1 \Big/ \text{Sqrt}\left[\left(\frac{e^{\frac{m*\omega*c^2}{\hbar}} * \sqrt{\pi}}{\sqrt{\frac{m*\omega}{\hbar}}}\right)\right]\right) * \text{Exp}[(m*\omega/\hbar)*(-x^2/2 - c*x)]$$ (*ground-state - left well*)

In[ ]:= Abs[Integrate[ψ0L[x] * ψ0L[x], {x, -Infinity, Infinity}]]
(*Checking the eigenfunction normalization:*)

Out[ ]= 1.

(*Plotting the eigenfunctions for node count:*)

In[ ]:=
```
Plot[{ψ0R[x], ψ1R[x], ψ0L[x]}, {x, -12 * 10^(-10), 12 * 10^(-10)},
 PlotLegends → "Expressions", PlotRange → All]
```

Out[ ]=
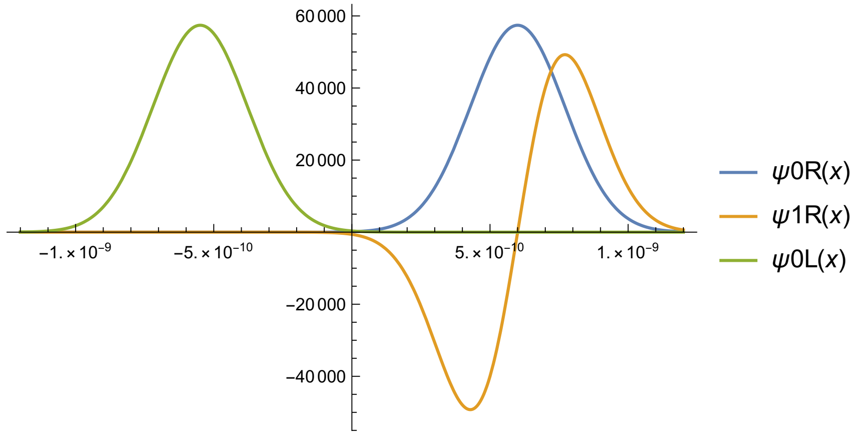


```
(*Normalizing and defining the split eigenfunctions:*)
```

In[ ]:=
```
Norm1 = Abs[1 / Sqrt[2 * (1 - Integrate[ψ0L[x] * ψ1R[x], {x, -Infinity, Infinity}])]]
Norm2 = Abs[1 / Sqrt[2 * (1 + Integrate[ψ0L[x] * ψ1R[x], {x, -Infinity, Infinity}])]]
```

Out[ ]= 0.707085

Out[ ]= 0.707128

In[ ]:=
```
χ1[x_] := Norm1 * (ψ0L[x] - ψ1R[x]) (*Lowest energy superposition state*)
```

In[ ]:=
```
χ2[x_] := Norm2 * (ψ0L[x] + ψ1R[x]) (*Highest energy superposition state*)
```

In[ ]:=
```
(*Plotting the eigenfunctions for node count:*)
Plot[{χ1[x], χ2[x]}, {x, -13 * 10^(-10), 13 * 10^(-10)},
 PlotLegends → "Expressions", PlotRange → All]
```

Out[ ]=
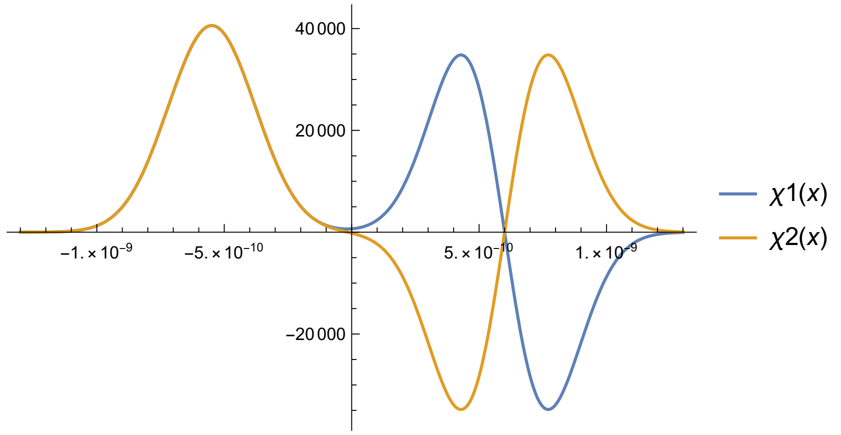


```
(*Variational calculation of the energy split eigenvalues:*)
```

In[ ]:=
```
NumberForm[E1 =
   Integrate[χ1[x] * (-(ħ ^ 2 / (2 * m)) * D[χ1[x], {x, 2}] + V[x] * χ1[x]), {x, -Infinity, Infinity}], 9]
NumberForm[E2 =
   Integrate[χ2[x] * (-(ħ ^ 2 / (2 * m)) * D[χ2[x], {x, 2}] + V[x] * χ2[x]), {x, -Infinity, Infinity}], 9]
```

Out[ ]//NumberForm=

$6.24741727 \times 10^{-19}$

Out[ ]//NumberForm=

$6.24835843 \times 10^{-19}$

In[ ]:=
```
(*Calculating the electronic coupling:*)
E2 - E0L
```

Out[ ]= 4.45929×10^{-23}

```
(*Calculating the tunneling time:*)
```

In[ ]:=
```
t = Pi * ħ / (E2 - E1)
```

Out[ ]= 3.51994×10^{-12}

Bacterial PRC: BL to HL

In[ ]:=
```
(*Defining the relevant physical constants (SI units):*)
```

In[ ]:=
```
ℏ = 1.0545 * 10 ^ (-34)
m = 9.1093 * 10 ^ (-31)
ω = 3.5 * 10 ^ (15)
a = 5.99013 * 10 ^ (-10)
b = ℏ * ω
c = Sqrt[a ^ 2 - (2 * ℏ / (m * ω))]
```

Out[ ]= 1.0545×10^{-34}

Out[ ]= 9.1093×10^{-31}

Out[ ]= 3.5×10^{15}

Out[ ]= 5.99013×10^{-10}

Out[ ]= 3.69075×10^{-19}

Out[ ]= 5.40988×10^{-10}

In[ ]:=
```
(*Defining the bistable potential:*)
```

In[ ]:=
```
V[x_] := Piecewise[{{(0.5) * (m * ω ^ 2) * (x + c) ^ 2 + b, x ≤ 0}, {((0.5) * m * ω ^ 2) * (x - a) ^ 2, x ≥ 0}}]
```

In[ ]:=
```
(*Graphically checking the form of the double well:*)
```

In[ ]:=
```
Plot[V[x], {x, -12 * 10 ^ (-10), 12 * 10 ^ (-10)}]
```

Out[ ]=

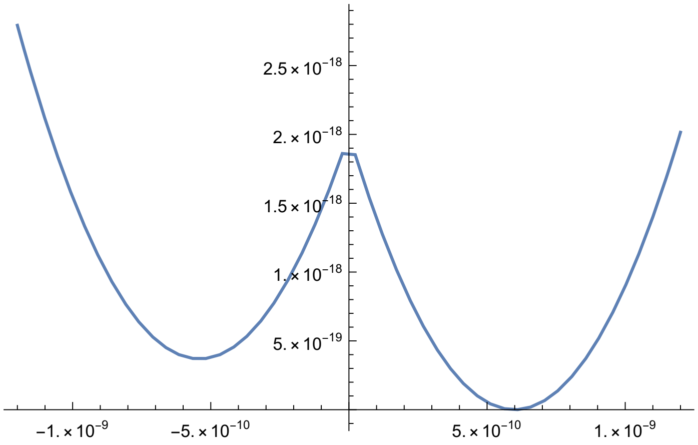

In[ ]:=
```
(*Comparing the energy values of simple
 oscillators with the height of the potential barrier:*)
```

In[ ]:=
```
NumberForm[E0R = ħ * ω / 2, 9]        (*right well*)
NumberForm[E1R = 3 * ħ * ω / 2 , 9]     (*right well*)
NumberForm[E0L = ħ * ω / 2 + ħ * ω, 9]  (*left well*)
```

Out[ ]//NumberForm=

1.845375×10^{-19}

Out[ ]//NumberForm=

5.536125×10^{-19}

Out[ ]//NumberForm=

5.536125×10^{-19}

In[ ]:=
```
V[0]  (*potential barrier*)
```

Out[ ]= 2.002×10^{-18}

In[ ]:=
```
(*Defining the eigenfunctions of simple harmonic oscillators:*)
```

In[ ]:=
```
ψ0R[x_] :=
```

$$\left(1 \Big/ \text{Sqrt}\left[\frac{e^{\frac{m*\omega*a^{\wedge}2}{\hbar}} * \sqrt{\pi}}{\sqrt{\frac{m*\omega}{\hbar}}}\right]\right) * \text{Exp}[(m*\omega/\hbar)*(-x^{\wedge}2/2 + a*x)] \text{ (*ground-state - right well*)}$$

In[ ]:=
```
Abs[Integrate[ψ0R[x] * ψ0R[x], {x, -Infinity, Infinity}]]
(*Checking the eigenfunction normalization:*)
```

Out[ ]= 1.

In[ ]:=

$$\psi 1R[x\_] := \left(1 \Big/ \text{Sqrt}\left[\frac{e^{\frac{m*\omega*a^{\wedge}2}{\hbar}} * m * \sqrt{\pi} * \omega^{\wedge}2}{\left(\frac{m*\omega}{\hbar}\right)^{3/2}}\right]\right) * 2 * \omega * \text{Sqrt}[m/2] * (x - a) *$$

$$\text{Exp}[(m*\omega/\hbar)*(-x^{\wedge}2/2 + a*x)] \text{ (*first excitited state - right well*)}$$

In[ ]:=
```
Abs[Integrate[ψ1R[x] * ψ1R[x], {x, -Infinity, Infinity}]]
(*Checking the eigenfunction normalization:*)
```

Out[ ]= 1.

In[ ]:=
```
ψ0L[x_] :=
```

$$\left(1 \Big/ \text{Sqrt}\left[\left(\frac{e^{\frac{m*\omega*c^{\wedge}2}{\hbar}} * \sqrt{\pi}}{\sqrt{\frac{m*\omega}{\hbar}}}\right)\right]\right) * \text{Exp}[(m*\omega/\hbar)*(-x^{\wedge}2/2 - c*x)] \text{ (*ground-state - left well*)}$$

In[ ]:=
```
Abs[Integrate[ψ0L[x] * ψ0L[x], {x, -Infinity, Infinity}]]
(*Checking the eigenfunction normalization:*)
```

Out[ ]= 1.

In[ ]:=
```
(*Plotting the eigenfunctions for node count:*)
```

In[ ]:=
```
Plot[{ψ0R[x], ψ1R[x], ψ0L[x]}, {x, -12 * 10 ^ (-10), 12 * 10 ^ (-10)},
 PlotLegends → "Expressions", PlotRange → All]
```

Out[ ]=

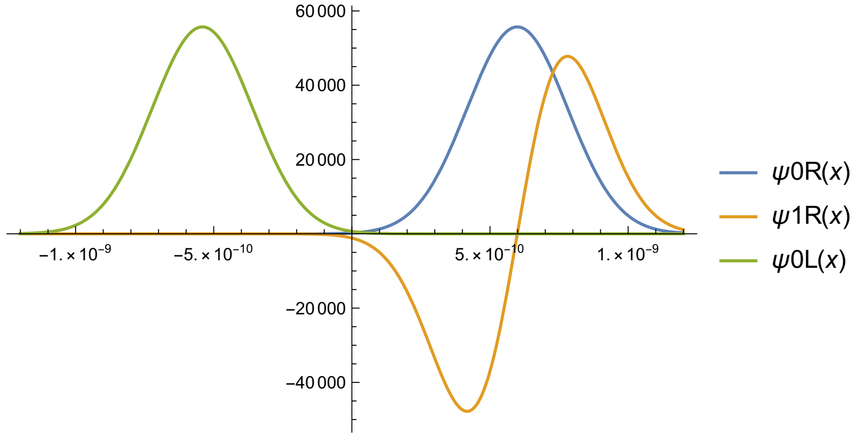

In[ ]:=
```
(*Normalizing and defining the split eigenfunctions:*)
```

In[ ]:=
```
Norm1 = Abs[1 / Sqrt[2 * (1 - Integrate[ψ0L[x] * ψ1R[x], {x, -Infinity, Infinity}])]]
Norm2 = Abs[1 / Sqrt[2 * (1 + Integrate[ψ0L[x] * ψ1R[x], {x, -Infinity, Infinity}])]]
```

Out[ ]= 0.707022

Out[ ]= 0.707192

In[ ]:=
```
χ1[x_] := Norm1 * (ψ0L[x] - ψ1R[x]) (*Lowest energy superposition state*)
```

In[ ]:=
```
χ2[x_] := Norm2 * (ψ0L[x] + ψ1R[x]) (*Highest energy superposition state*)
```

In[ ]:=
```
(*Plotting the eigenfunctions for node count:*)
Plot[{χ1[x], χ2[x]}, {x, -13 * 10 ^ (-10), 13 * 10 ^ (-10)},
 PlotLegends → "Expressions", PlotRange → All]
```

Out[ ]=

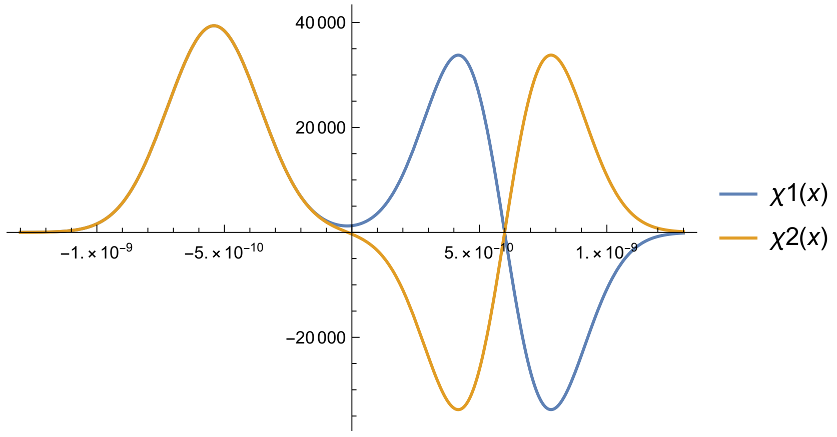

In[ ]:=
```
(*Variational calculation of the energy split eigenvalues:*)
```

In[ ]:=
```
NumberForm[E1 =
   Integrate[χ1[x] * (-(ħ ^ 2 / (2 * m)) * D[χ1[x], {x, 2}] + V[x] * χ1[x]), {x, -Infinity, Infinity}], 9]
NumberForm[E2 =
   Integrate[χ2[x] * (-(ħ ^ 2 / (2 * m)) * D[χ2[x], {x, 2}] + V[x] * χ2[x]), {x, -Infinity, Infinity}], 9]
```

Out[ ]//NumberForm=

$5.5345087 \times 10^{-19}$

Out[ ]//NumberForm=

$5.53756423 \times 10^{-19}$

In[ ]:=
```
(*Calculating the electronic coupling:*)
E2 - E0L
```

Out[ ]= 1.43923×10^{-22}

In[ ]:=
```
(*Calculating the tunneling time:*)
```

In[ ]:=
```
t = Pi * ħ / (E2 - E1)
```

Out[ ]= 1.0842×10^{-12}

Bacterial PRC: HL to QA

```
In[ ]:= (*Defining the relevant physical constants (SI units):*)

In[ ]:= ℏ = 1.0545 * 10 ^ (-34)
        m = 9.1093 * 10 ^ (-31)
        ω = 1.97 * 10 ^ (15)
        a = 9.71256 * 10 ^ (-10)
        b = ℏ * ω
        c = Sqrt[a ^ 2 - (2 * ℏ / (m * ω))]
```

Out[ ]= 1.0545×10^{-34}

Out[ ]= 9.1093×10^{-31}

Out[ ]= 1.97×10^{15}

Out[ ]= 9.71256×10^{-10}

Out[ ]= 2.07737×10^{-19}

Out[ ]= 9.08743×10^{-10}

```
In[ ]:= (*Defining the bistable potential:*)

In[ ]:= V[x_] := Piecewise[{{(0.5) * (m * ω ^ 2) * (x + c) ^ 2 + b, x ≤ 0}, {((0.5) * m * ω ^ 2) * (x - a) ^ 2, x ≥ 0}}]

In[ ]:= (*Graphically checking the form of the double well:*)

In[ ]:= Plot[V[x], {x, -18 * 10 ^ (-10), 18 * 10 ^ (-10)}]
```

Out[ ]=

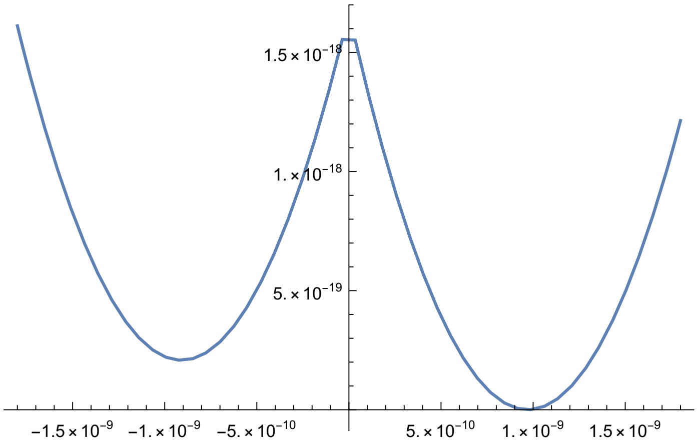


```
In[ ]:= (*Comparing the energy values of simple
         oscillators with the height of the potential barrier:*)
```

In[ ]:=
```
NumberForm[E0R = ℏ * ω / 2, 9]         (*right well*)
NumberForm[E1R = 3 * ℏ * ω / 2 , 9]     (*right well*)
NumberForm[E0L = ℏ * ω / 2 + ℏ * ω, 9]  (*left well*)
```

Out[ ]//NumberForm=

$1.0386825 \times 10^{-19}$

Out[ ]//NumberForm=

$3.1160475 \times 10^{-19}$

Out[ ]//NumberForm=

$3.1160475 \times 10^{-19}$

In[ ]:= `V[0] (*potential barrier*)`

Out[ ]= 1.66746×10^{-18}

In[ ]:= `(*Defining the eigenfunctions of simple harmonic oscillators:*)`

In[ ]:= ψ0R[x_] :=

$$\left(1 \Big/ \mathrm{Sqrt}\left[\frac{e^{\frac{m*\omega*a^{\wedge}2}{\hbar}} * \sqrt{\pi}}{\sqrt{\frac{m*\omega}{\hbar}}}\right]\right) * \mathrm{Exp}[(m*\omega/\hbar)*(-x^{\wedge}2/2+a*x)]\ \text{(*ground-state - right well*)}$$

In[ ]:=
```
Abs[Integrate[ψ0R[x] * ψ0R[x], {x, -Infinity, Infinity}]]
(*Checking the eigenfunction normalization:*)
```

Out[ ]= 1.

In[ ]:=

$$\psi 1R[x\_] := \left(1 \Big/ \mathrm{Sqrt}\left[\frac{e^{\frac{m*\omega*a^{\wedge}2}{\hbar}} * m * \sqrt{\pi} * \omega^{\wedge}2}{\left(\frac{m*\omega}{\hbar}\right)^{3/2}}\right]\right) * 2 * \omega * \mathrm{Sqrt}[m/2] * (x-a) *$$

$$\mathrm{Exp}[(m*\omega/\hbar)*(-x^{\wedge}2/2+a*x)]\ \text{(*first excitited state - right well*)}$$

In[ ]:=
```
Abs[Integrate[ψ1R[x] * ψ1R[x], {x, -Infinity, Infinity}]]
(*Checking the eigenfunction normalization:*)
```

Out[ ]= 1.

In[ ]:= ψ0L[x_] :=

$$\left(1 \Big/ \mathrm{Sqrt}\left[\left(\frac{e^{\frac{m*\omega*c^{\wedge}2}{\hbar}} * \sqrt{\pi}}{\sqrt{\frac{m*\omega}{\hbar}}}\right)\right]\right) * \mathrm{Exp}[(m*\omega/\hbar)*(-x^{\wedge}2/2-c*x)]\ \text{(*ground-state - left well*)}$$

In[ ]:=
```
Abs[Integrate[ψ0L[x] * ψ0L[x], {x, -Infinity, Infinity}]]
(*Checking the eigenfunction normalization:*)
```

Out[ ]= 1.

In[ ]:= `(*Plotting the eigenfunctions for node count:*)`

In[ ]:=
```
Plot[{ψ0R[x], ψ1R[x], ψ0L[x]}, {x, -18 * 10 ^ (-10), 18 * 10 ^ (-10)},
  PlotLegends → "Expressions", PlotRange → All]
```

Out[ ]=

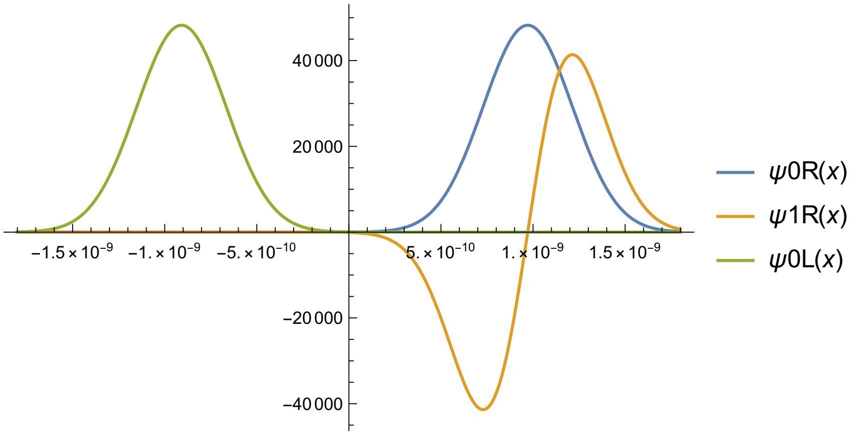

In[ ]:=
```
(*Normalizing and defining the split eigenfunctions:*)
```

In[ ]:=
```
Norm1 = Abs[1 / Sqrt[2 * (1 - Integrate[ψ0L[x] * ψ1R[x], {x, -Infinity, Infinity}])]]
Norm2 = Abs[1 / Sqrt[2 * (1 + Integrate[ψ0L[x] * ψ1R[x], {x, -Infinity, Infinity}])]]
```

Out[ ]= 0.707106

Out[ ]= 0.707107

In[ ]:=
```
χ1[x_] := Norm1 * (ψ0L[x] - ψ1R[x]) (*Lowest energy superposition state*)
```

In[ ]:=
```
χ2[x_] := Norm2 * (ψ0L[x] + ψ1R[x]) (*Highest energy superposition state*)
```

In[ ]:=
```
(*Plotting the eigenfunctions for node count:*)
Plot[{χ1[x], χ2[x]}, {x, -18 * 10 ^ (-10), 18 * 10 ^ (-10)},
  PlotLegends → "Expressions", PlotRange → All]
```

Out[ ]=

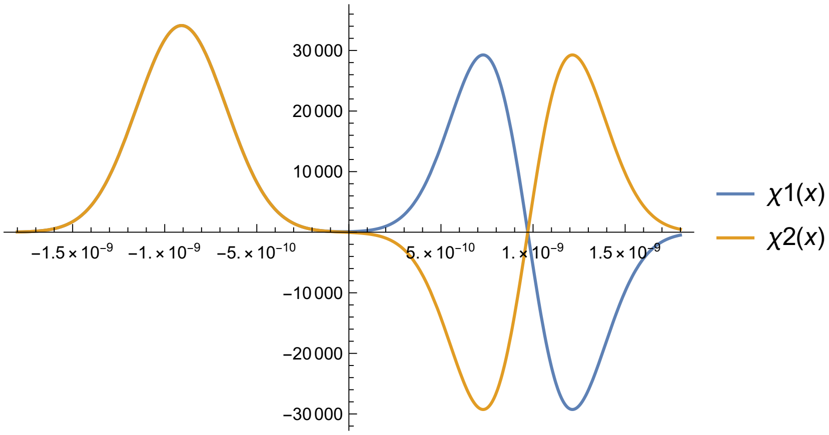

In[ ]:=
```
(*Variational calculation of the energy split eigenvalues:*)
```

```
In[ ]:= NumberForm[E1 =
    Integrate[χ1[x] * (-(ħ^2/(2*m)) * D[χ1[x], {x, 2}] + V[x] * χ1[x]), {x, -Infinity, Infinity}], 9]
  NumberForm[E2 =
    Integrate[χ2[x] * (-(ħ^2/(2*m)) * D[χ2[x], {x, 2}] + V[x] * χ2[x]), {x, -Infinity, Infinity}], 9]
```

Out[ ]//NumberForm=

$3.11603997 \times 10^{-19}$

Out[ ]//NumberForm=

$3.11605442 \times 10^{-19}$

```
In[ ]:= (*Calculating the electronic coupling:*)
  E2 - E0L
```

Out[ ]= 6.91922×10^{-25}

```
In[ ]:= (*Calculating the tunneling time:*)
```

```
In[ ]:= t = Pi * ħ / (E2 - E1)
```

Out[ ]= 2.29215×10^{-10}

DNA Photolyase: single-step electron transfer to 3’-CPD

```
(*Defining the relevant physical constants (SI units):*)
```

In[ ]:=
```
ℏ = 1.054572 * 10 ^ (-34)
m = 9.11 * 10 ^ (-31)
ω = 6.38 * 10 ^ 15
a = 5.62 * 10 ^ (-10)
b = ℏ * ω
c = Sqrt[a ^ 2 - (2 * b / (m * ω ^ 2))]
```

Out[ ]= 1.05457×10^{-34}

Out[ ]= 9.11×10^{-31}

Out[ ]= 6.38×10^{15}

Out[ ]= 5.62×10^{-10}

Out[ ]= 6.72817×10^{-19}

Out[ ]= 5.2873×10^{-10}

```
(*Defining the bistable potential:*)
```

In[ ]:=
```
V[x_] := Piecewise[{{(0.5) * (m * ω ^ 2) * (x + c) ^ 2 + b, x ≤ 0}, {((0.5) * m * ω ^ 2) * (x - a) ^ 2, x ≥ 0}}]
```

```
(*Graphically checking the form of the double well:*)
```

In[ ]:=
```
Plot[V[x], {x, -12 * 10 ^ (-10), 12 * 10 ^ (-10)}]
```

Out[ ]=

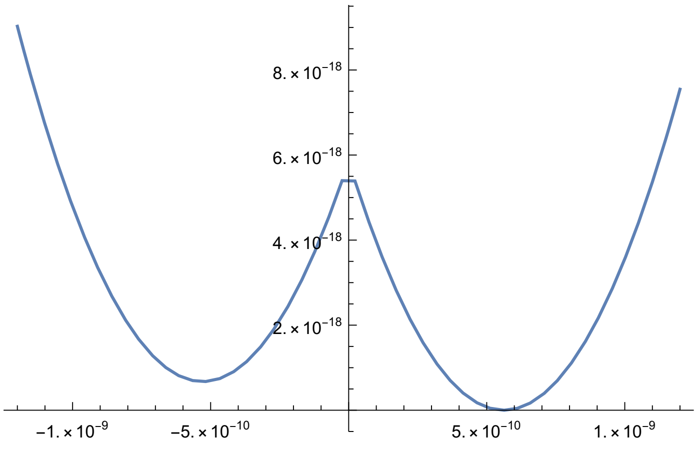


```
(*Comparing the energy values of simple
 oscillators with the height of the potential barrier:*)
```

```
NumberForm[E0R = ℏ * ω / 2, 10]           (*right well*)
NumberForm[E1R = 3 * ℏ * ω / 2, 10]        (*right well*)
NumberForm[E0L = ℏ * ω / 2 + ℏ * ω, 10]   (*left well*)
```

Out[ ]//NumberForm=

$3.36408468 \times 10^{-19}$

Out[ ]//NumberForm=

$1.009225404 \times 10^{-18}$

Out[ ]//NumberForm=

$1.009225404 \times 10^{-18}$

In[ ]:= `V[0]  (*potential barrier*)`

Out[ ]= 5.85602×10^{-18}

```
(*Defining the eigenfunctions of simple harmonic oscillators:*)
```

`ψ0R[x_] :=`

$$\left(1 \Big/ \text{Sqrt}\left[\frac{e^{\frac{m*\omega*a^2}{\hbar}} * \sqrt{\pi}}{\sqrt{\frac{m*\omega}{\hbar}}}\right]\right) * \text{Exp}[(m*\omega/\hbar)*(-x^2/2 + a*x)]$$ (*ground-state - right well*)

In[ ]:= `Abs[Integrate[ψ0R[x] * ψ0R[x], {x, -Infinity, Infinity}]]`
`(*Checking the eigenfunction normalization:*)`

Out[ ]= 1.

$$\psi 1R[x\_] := \left(1 \Big/ \text{Sqrt}\left[\frac{e^{\frac{m*\omega*a^2}{\hbar}} * m * \sqrt{\pi} * \omega^2}{\left(\frac{m*\omega}{\hbar}\right)^{3/2}}\right]\right) * 2 * \omega * \text{Sqrt}[m/2] * (x - a) *$$

$$\text{Exp}[(m*\omega/\hbar)*(-x^2/2 + a*x)]$$ (*first excitited state - right well*)

In[ ]:= `Abs[Integrate[ψ1R[x] * ψ1R[x], {x, -Infinity, Infinity}]]`
`(*Checking the eigenfunction normalization:*)`

Out[ ]= 1.

In[ ]:= `ψ0L[x_] :=`

$$\left(1 \Big/ \text{Sqrt}\left[\left(\frac{e^{\frac{m*\omega*c^2}{\hbar}} * \sqrt{\pi}}{\sqrt{\frac{m*\omega}{\hbar}}}\right)\right]\right) * \text{Exp}[(m*\omega/\hbar)*(-x^2/2 - c*x)]$$ (*ground-state - left well*)

In[ ]:= `Abs[Integrate[ψ0L[x] * ψ0L[x], {x, -Infinity, Infinity}]]`
`(*Checking the eigenfunction normalization:*)`

Out[ ]= 1.

```
(*Plotting the eigenfunctions for node count:*)
```

In[ ]:= Plot[{ψ0R[x], ψ1R[x], ψ0L[x]}, {x, -10 * 10 ^ (-10), 10 * 10 ^ (-10)},
 PlotLegends → "Expressions", PlotRange → All]

Out[ ]=
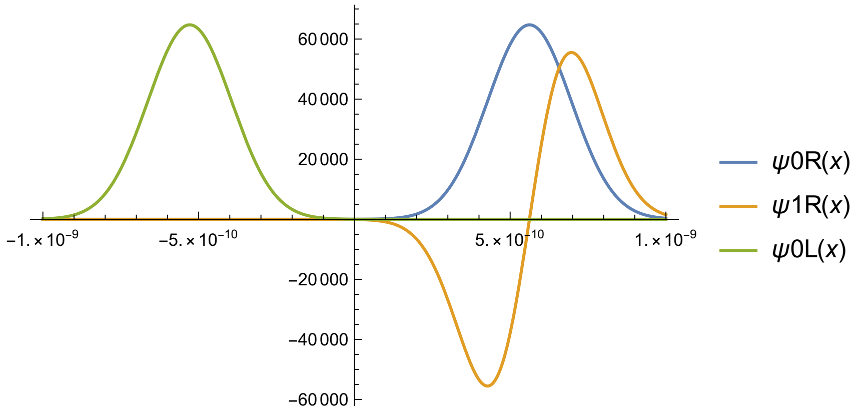

(*Normalizing and defining the split eigenfunctions:*)

In[ ]:= Norm1 = Abs[1 / Sqrt[2 * (1 - Integrate[ψ0L[x] * ψ1R[x], {x, -Infinity, Infinity}])]]
Norm2 = Abs[1 / Sqrt[2 * (1 + Integrate[ψ0L[x] * ψ1R[x], {x, -Infinity, Infinity}])]]

Out[ ]= 0.707107

Out[ ]= 0.707107

χ1[x_] := Norm1 * (ψ0L[x] - ψ1R[x]) (*Lowest energy superposition state*)

χ2[x_] := Norm2 * (ψ0L[x] + ψ1R[x]) (*Highest energy superposition state*)

In[ ]:= (*Plotting the eigenfunctions for node count:*)
Plot[{χ1[x], χ2[x]}, {x, -10 * 10 ^ (-10), 10 * 10 ^ (-10)},
 PlotLegends → "Expressions", PlotRange → All]

Out[ ]=
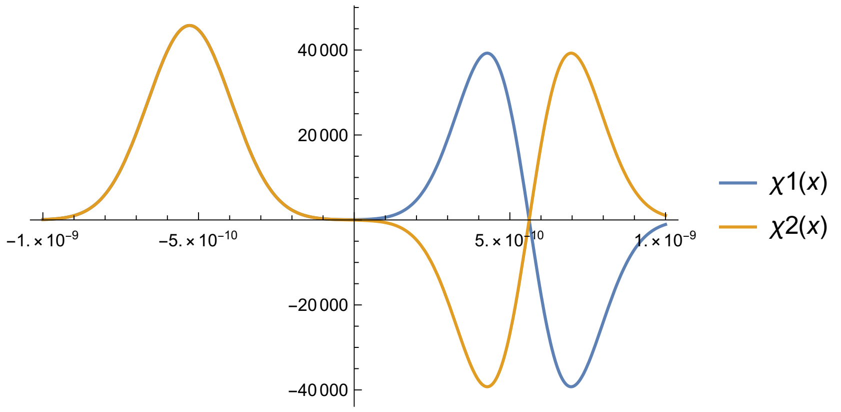

(*Variational calculation of the energy split eigenvalues:*)

```
In[ ]:= NumberForm[E1 =
          Integrate[χ1[x] * (-(ℏ^2 / (2 * m)) * D[χ1[x], {x, 2}] + V[x] * χ1[x]), {x, -Infinity, Infinity}], 9]
        NumberForm[E2 =
          Integrate[χ2[x] * (-(ℏ^2 / (2 * m)) * D[χ2[x], {x, 2}] + V[x] * χ2[x]), {x, -Infinity, Infinity}], 9]
```

Out[ ]//NumberForm=

$1.00922472 \times 10^{-18}$

Out[ ]//NumberForm=

$1.00922604 \times 10^{-18}$

```
In[ ]:= (*Calculating the electronic coupling:*)
        E2 - E0L
```

Out[ ]= 6.32502×10^{-25}

```
(*Calculating the tunneling time:*)

In[ ]:= t = Pi * ℏ / (E2 - E1)
```

Out[ ]= 2.51415×10^{-10}
